# Mesophilic Anaerobic Digestion as a Pre-Treatment to Enhance the Composting Biodegradation of PLA-Based Compostable Packaging

**DOI:** 10.3390/polym18212691

**Published:** 2026-10-30

**Authors:** Gaël Huet, Julie Lamarque, Florian Monlau, Valentin Puchelle, Cecilia Sambusiti, Juliana Catalina Suarez Murcia, Sandra Domenek, Emmanuelle Gastaldi

**Affiliations:** 1 UMR IATE, Université de Montpellier, INRAE, L’institut Agro Montpellier, 34000 Montpellier, France; 2 TotalEnergies, OneTech R&D, PERL—Pôle Economique 2, 64170 Lacq, France; 3 UMR IPREM, Université de Pau et Des Pays de l’Adour, CNRS, 64053 Pau, France; 4 TotalEnergies, OneTech Belgium, Feluy—Zone Industrielle C, 7181 Seneffe, Belgium; 5 TotalEnergies, OneTech R&D, CSTJF—Avenue Larribau, 64018 Pau, France; 6 UMR SayFood, Université Paris-Saclay, INRAE, AgroParisTech, 91120 Palaiseau, France

**Keywords:** hydrolysis, polymer degradation, molecular weight reduction, fragmentation, biodegradation kinetics, PLA-based materials

## Abstract

The limited biodegradation of many commercially available compostable polymer formulations under mesophilic anaerobic digestion (AD) currently represents one of the main barriers to their acceptance in integrated organic recycling systems. This study evaluated whether mesophilic anaerobic digestion, followed by industrial composting, can improve the subsequent biodegradation of these materials. Compostable coffee capsules made of polylactic acid (PLA), poly(hydroxybutyrate-*co*-valerate) (PHBV), poly(butylene adipate-*co*-terephthalate) (PBAT), and poly(butylene succinate) (PBS) were used as model materials to evaluate physicochemical and morphological changes induced by AD. Mesophilic AD significantly altered the supramolecular structure of PLA-, PHBV-, and PBAT-based capsules, whereas PBS-rich formulations showed limited macroscopic changes. AD promoted PLA hydrolysis, induced chain scission, reduced polymer molecular weight, and triggered material fragmentation. These structural modifications markedly accelerated the subsequent biodegradation of PLA-based capsules under industrial composting conditions, reducing the half-time by a factor of 2.3–3.7. Integrating mesophilic AD with composting therefore appears to be a promising strategy for treating biowaste containing industrially compostable packaging. These results demonstrate that the response of commercial compostable formulations to anaerobic digestion is formulation-dependent and governed by polymer-specific structural changes. The study highlights the importance of polymer formulation in determining biodegradation pathways under integrated organic recycling conditions.

## 1. Introduction

Compostable packaging increasingly relies on multicomponent polyester formulations combining poly(lactic acid) (PLA), poly(butylene adipate-*co*-terephthalate) (PBAT), poly(butylene succinate) (PBS) and poly(hydroxybutyrate-*co*-valerate) (PHBV), to balance processability, mechanical properties and biodegradation performance. These materials are intended to accompany food waste throughout biological treatment, thereby facilitating the transition toward circular bio-waste management [1,2]. When co-collected with food waste, these materials enter anaerobic digestion (AD) or industrial composting facilities intended to produce organic amendments, either following biogas production or via composting alone. The management of bio-waste and compostable packaging has become a major focus of European waste policy, with the recent implementation of mandatory separate collection of bio-waste from 2024 [3,4].

Among compostable packaging items, coffee capsules represent a particularly relevant case study. These single-use products are widely consumed and frequently enter biowaste streams together with coffee grounds, making them common inputs to anaerobic digestion facilities in countries where certified compostable packaging is accepted with food waste. In addition, their formulations typically combine several biodegradable polyesters including PLA, PHBV, PBAT, and PBS, making them representative of the diverse polymer blends currently used in commercial compostable packaging.

However, not all certified compostable materials biodegrade effectively under mesophilic AD, which is currently the most widespread treatment technology. While polyhydroxyalkanoates (PHAs) and starch-based blends often show satisfactory biodegradation, some polyesters, including PLA, PBAT and PBS, exhibit slower degradation rates due to their limited enzymatic accessibility and hydrolysis resistance [5,6,7]. This incomplete degradation can lead to polymer residues in the digestate, raising quality concerns for its subsequent agricultural use.

Combining AD with subsequent industrial composting has recently emerged as a promising strategy to improve the overall degradation efficiency of bio-based biodegradable materials [8,9]. Recent studies have investigated sequential anaerobic digestion and composting of commercial compostable products, demonstrating the potential of the combined treatment while highlighting the influence of polymer formulation and processing conditions on biodegradation [10,11]. The physicochemical and biological conditions prevailing during AD can induce transformations of biodegradable polymers, including hydrolysis, chain scission, and fragmentation [12,13]. Such AD-induced modifications may facilitate subsequent biodegradation under industrial composting conditions [14]. However, the relationship between molecular changes induced by mesophilic AD and subsequent composting biodegradation kinetics remains poorly understood, particularly across different commercial polymer formulations. In this context, understanding how AD modifies such materials is essential for assessing their compatibility with treatment chains combining anaerobic digestion followed by composting increasingly implemented in European biowaste management systems.

This study investigates how mesophilic AD alters the structure and physicochemical properties of commercial compostable packaging materials and how these changes influence their subsequent biodegradation kinetics under industrial composting conditions.

## 2. Materials and Methods

### 2.1. Materials and Analytical Methods

Four commercially available compostable coffee capsules (referred to as Cap1-Cap4) were selected for this study. The capsules were selected from a broader set of 12 commercial coffee capsules previously characterized in our laboratory [15] to cover different polymer formulations showing incomplete biodegradation under mesophilic anaerobic digestion. PHBV-based capsules, which showed high anaerobic biodegradability (>90%), were not included. No information on polymer composition was disclosed by the manufacturers, including the identity of the major polymer. The compositions reported here were therefore experimentally determined in our laboratory as described below. All were certified as industrially compostable according to the EN 13432 standard [16]. For confidentiality reasons, the trademarks and certificate holders are not disclosed. The capsules were unused, emptied, rinsed with deionized water, and dried at 38 °C before analysis.

Total Solids (TS) and Volatile Solids (VS) were determined in triplicate by loss on drying at 105 °C and loss on ignition at 550 °C, respectively [17]. The contents of organic carbon (C), hydrogen (H), nitrogen (N) and sulphur (S) were measured in duplicate with an elemental analyzer (Vario Micro Cube, Elementar, DE), while the oxygen content (O) was estimated as the difference between VS and C, H, N and S contents. The polymer composition (wt%) was determined using a combination of thermogravimetric analysis (TGA) and liquid-state proton nuclear magnetic resonance spectroscopy (^1^H NMR) according to the method proposed by [18]. TGA was performed under nitrogen (10 °C/min, 25–600 °C) to assess thermal stability, and DTG peaks were used to distinguish the thermal degradation profiles of the individual polymers. T_max_ values corresponded to the temperature at maximum weight loss rate for each polymer. TGA curves and their respective derivatives were used to quantify the mass fraction of each component. Polymer composition was further validated by confirming the chemical nature of the recovered polymer residues (PLA, PHBV, PBAT and PBS) by ^1^H NMR spectra as described by [19]. As none of the four capsules contained starch or other components producing overlapping thermal degradation profiles that prevented direct quantification by TGA, ^1^H NMR integration was not used to calculate the polymer mass fractions reported in this study. Size exclusion chromatography (SEC) analyses were performed using an Agilent 1260 Infinity II HPLC System (Agilent Technologies, Santa Clara, CA, USA) equipped with a KF-G 4A column guard (10 mm × 4.6 mm), KF-804L and KF-803L Columns (300 × 8 mm) (Shodex, Resonac Corporation, Tokyo, Japan), connected in series and thermoregulated at 30 °C. Detection was carried out with an Agilent 1290 Infinity II refractometric detector and UV detector with dual mode at 254 and 280 nm (Agilent Technologies, Santa Clara, CA, USA). Material samples, including the solid polymer residues recovered after AD and separated from the digestion medium, were completely solubilized in CHCl_3_ and filtered through 0.45 µm PTFE filters prior to SEC analysis. No insoluble polymer fraction remained after dissolution. The concentration of the samples was 5 g/L, and the flow rate was 1 mL/min. Molecular weights were calibrated using polystyrene standards. The number-average molecular weight (M_n_), weight-average molecular weight (M_w_), and dispersity index (ĐM = M_w_/M_n_) were determined from SEC chromatograms. Differential scanning calorimetry (DSC) analyses were performed to evaluate changes in polymer thermal behavior and crystallinity before and after AD treatment using a DSC Q200 (TA Instruments, New Castle, DE, USA) under a nitrogen atmosphere. Approximately 5 mg of each sample was sealed in aluminum pans and subjected to a heating–cooling–heating cycle at a rate of 10 °C/min. The first heating scan was carried out from −40 °C to 200 °C, followed by cooling from 200 °C to −60 °C and a second heating scan from −60 °C to 200 °C. The degree of crystallinity (X_c_) was estimated from the melting enthalpy (ΔH_m_) and cold crystallization enthalpy (ΔH_cc_), considering the mass fraction of the corresponding polymer in each formulation. The theoretical melting enthalpies of fully crystalline PLA, PHBV, PBS, and PBAT were taken as 93, 146, 110.3, and 114 J g^−1^, respectively [20,21,22,23]. Due to overlapping and distorted melting endotherms, the assignment of thermal transitions to individual polymer components was tentative, and the resulting crystallinity values were considered estimates. Surface morphology was observed using a Scanning Electron Microscope Phenom ProX (Phenom-World B.V., Eindhoven, The Netherlands) before and after AD following the procedure described by [24].

### 2.2. Anaerobic Digestion Tests

Biochemical methane potential (BMP) tests were performed under mesophilic conditions to assess the anaerobic degradability of the coffee capsules. The assays followed the guidelines of the German Biomass Research Centre (DBFZ) [25] and the EN ISO 13975 standard [26]. Three BMP series were carried out over one year, using inoculum from the same industrial anaerobic digester treating biowaste and agricultural residues. Before each series, the inoculum was stabilized at 38 °C until biogas production ceased. Across all series, its characteristics were: pH = 8.0 ± 0.1, TS = 6.5 ± 1.0% *w*/*w*, VS = 58 ± 3% TS, FOS/TAC = 0.16 ± 0.01, and N-NH_4_^+^ = 2.7 ± 0.2 g L^−1^. Coffee capsules were cut as 1 cm × 1 cm pieces, and triplicate 500-mL serum bottles were prepared at an inoculum-to-substrate ratio of 0.37 gVSproduct/gVS inoculum [27]. Macronutrient and micronutrient solutions and NaHCO_3_ buffer were added to ensure suitable microbial activity. Distilled water was used to reach a working volume of ~200 mL and an organic loading rate of 30–40 gVS/L. Each series included duplicate blanks (inoculum only) and duplicate positive controls with microcrystalline cellulose (CMC) to validate test performance according to DBFZ and EN ISO 13975 criteria [25,26]. Bottles were flushed with N_2_, sealed, and incubated at 38 °C with continuous stirring (75 rpm) for 105 days. Methane production was monitored by periodic headspace pressure measurements (LEO 2, KELLER AG, Winterthur, Switzerland) and gas chromatography MicroGC 990 (Agilent Technologies, Santa Clara, CA, USA) equipped with Molsieve 5 Å and PoraPLOT U columns. Cumulative methane production was normalized to standard temperature and pressure (0 °C, 101,325 Pa) and expressed on a dry gas basis. Methane production from blanks was subtracted from all experimental values. The experimental BMP (BMP_exp_) was defined as the maximum methane yield obtained during the test, and the reported values correspond to the mean of three replicate reactors incubated for 105 days. Biodegradability (BI) was calculated as the ratio between the normalized BMP_exp_ and the BMP_th_ of each product, where BMP_th_ was estimated from elemental composition using the Buswell–Boyle equation [28]. At the end of AD, the solid residues were recovered, washed with distilled water, and air-dried prior to further analyses (composition, SEC, TGA, and composting tests).

### 2.3. Composting Biodegradation Tests

Aerobic biodegradation tests were carried out under simulated industrial composting conditions according to ISO 14855-1 [29]. AD residues and untreated materials were mixed with mature compost (1:6 *w*/*w*) and incubated in 1 L aerated reactors at 58 ± 1 °C for 90 days. A relative humidity of 50 to 55% was maintained continuously in the reactors by regularly adding water. The emitted CO_2_ was trapped in 0.2 M NaOH and quantified by titration with 0.1 M HCl. Biodegradation (%) was calculated from the cumulative CO_2_ released by the test material relative to the theoretical CO_2_ yield based on its elemental composition. All experimental details, including reactor design, aeration system, and CO_2_ quantification, followed the procedures described in [24]. Blanks containing compost alone were used to correct background CO_2_ production. The biodegradation curves were expressed as the mean ± standard deviation of the three experimental replicates. Biodegradation kinetics were fitted with the Hill model:

$$\mathrm{Deg}=\;\frac{{\mathrm{Deg}}_{\max}\;\times \;t^{n}}{k^{n}\;+\;t^{n}}$$

where *Deg_max_* is the maximum degradation extent (%), *k* the half-degradation time (days), and *n* is the shape parameter controlling the steepness of the sigmoid function. The Hill model was fitted independently to each of the three experimental replicates using nonlinear least-squares regression (OriginPro 2023, OriginLab Corporation, Northampton, MA, USA), considering all available time points, including day 91 where available. Differences in half-degradation times (*k*) between untreated and AD-pretreated samples were evaluated using Welch’s *t*-test, with a significance threshold of *p* < 0.05. Given the small sample size, statistical comparisons were interpreted cautiously.

## 3. Results and Discussion

The influence of mesophilic anaerobic digestion (AD) on the physicochemical, structural, and biodegradation behavior of compostable packaging materials was analyzed to determine how this pre-treatment affects their subsequent industrial composting performance. The following sections address (i) compositional and morphological changes induced during the AD process, (ii) resulting modifications in molecular weight and thermal stability, and (iii) their impact on aerobic biodegradation kinetics under industrial composting conditions.

### 3.1. Changes in Polymer Composition After Mesophilic Anaerobic Digestion

To better understand the results from the degradation experiments, the composition of the four coffee capsules was initially characterized. Table 1 shows that all coffee capsules contained PLA blended with another polymer: PHBV in Cap1, PBAT in Cap2, and PBS in Cap3, while Cap4 was made of pure PLA. Cap1 and Cap2 were predominantly composed of PLA, representing about 60% and 70% of their formulation, respectively, whereas Cap3 differed markedly, being mainly composed of PBS (≈52%) and containing only about 30% PLA. For Cap3, the decrease in the relative PLA fraction from 30.3 to 25.8 wt.% is consistent with preferential PLA alteration or removal during AD. However, these relative compositional changes do not allow the absolute extent of PLA degradation to be quantified.

Cap3 exhibited the highest inorganic fraction (17.7 ± 2.3 wt.%). ATR-FTIR analysis revealed absorption bands at approximately 670 and 1010 cm^−1^, consistent with characteristic silicate vibrations of talc [30] (Figure S1), suggesting that talc may be the predominant mineral filler in Cap3. Talc may act as a nucleating agent, potentially influencing polymer crystallinity.

The cumulative methane production kinetics (Figure S2) showed that methane production was still ongoing after 105 days of mesophilic AD, without reaching a clear plateau for any of the four capsules. After 105 days of mesophilic AD, their behavior varied markedly (Figure 1). Cap1 achieved the highest methane yield (BMP = 296 NmLCH_4_/gVS) and biodegradability index (BI = 59%), while Cap2 and Cap3 exhibited limited performance (BMP ≤ 40 NmLCH_4_/gVS; BI ≤ 7%). Cap4 showed intermediate values (BMP = 137 NmLCH_4_/gVS; BI = 29%). Although few studies have focused on coffee capsules, other authors also reported rather low biodegradation performance (BMP = 67–127 NmLCH_4_/gVS; BI = 12–28%) after 100 days of mesophilic AD for PLA-based capsules of unspecified composition [31], supporting the limited degradability of such materials under anaerobic conditions.

Comparison of coffee capsule composition before and after AD (Table 1) identified the polymers potentially undergoing preferential biodegradation. In Cap1, PHBV content decreased by 13%, while PLA increased by 11%, indicating partial PHBV biodegradation consistent with its relatively high biodegradability under mesophilic AD [5,8,15,32]. The persistence of about 26% PHBV suggests incomplete conversion within the 105-day digestion period. The high BI of Cap1 also implies partial PLA degradation. In Cap2 and Cap3, PLA content decreased (−13% and −4.5%, respectively) while PBAT and PBS contents increased (+12% and +4.3%), consistent with their limited biodegradation under AD [7,12,13,15,19]. The low BI values (<10%) of these samples, compared with the PLA control (Cap4, BI = 29%), further support that PLA can undergo partial biodegradation under mesophilic AD conditions [32,33,34].

The degradation of the polyesters found in these capsules may involve both abiotic factors (e.g., temperature and hydrolysis) and biotic processes, potentially leading to microbial assimilation and mineralization of degradation products [35]. Although the specific microbial communities involved in the present study were not characterized, the recent literature provides insights into the mechanisms potentially involved. For example, PHAs (such as PHBV in Cap1) contain hydrolysable ester linkages that can be cleaved by microbial enzymes, including extracellular PHA depolymerases produced by certain anaerobic microorganisms [36].

By contrast, the PLA degradation (present in all capsules) is limited under mesophilic AD conditions [5,7], partly due to the glassy state of its amorphous phase, which restricts chain mobility and slows hydrolysis as reported by Limsukon et al. [37]. Hydrolysis may be facilitated by increased chain mobility near or above the glass transition temperature (Tg), or by water-induced plasticization. The reduction in PLA molecular weight observed in the present study may thus be primarily attributed to abiotic hydrolysis of ester bonds. The resulting lower-molecular-weight degradation products may subsequently become available for microbial assimilation and mineralization, although these processes were not directly investigated.

PBAT and PBS (present in Cap2 and Cap3, respectively) generally exhibit limited biodegradation under mesophilic AD conditions. For PBAT, the presence of aromatic terephthalate units and its structural organization may restrict polymer accessibility and subsequent biodegradation. Moreover, Poulsen et al. [38] demonstrated under thermophilic conditions that microbial assimilation of individual PBAT degradation products differs considerably, with terephthalic acid showing particularly limited assimilation. PBS may also exhibit restricted biodegradation under mesophilic AD, depending on its molecular and morphological characteristics.

Finally, polymer composition, additives, and material morphology may strongly influence degradation behavior, as highlighted by previous studies on commercial compostable products [39,40]. In addition to these material-related factors, abiotic processes may also contribute substantially to the observed mass loss during anaerobic digestion, as demonstrated by Ferreri et al. [41] for commercial compostable bags, although their polymer composition was not experimentally characterized.

### 3.2. Morphological and Structural Changes After Anaerobic Digestion

Visual and SEM observations of coffee capsules before and after mesophilic AD treatment (Figure 2) revealed strong surface erosion and fragmentation of PLA/PHBV-, PLA/PBAT-, and PLA-based capsules, whereas the PBS/PLA capsule (Cap3) retained its macroscopic integrity. Cap1 (PLA/PHBV) lost its physical integrity through surface erosion and numerous small cracks, while Cap2 (PLA/PBAT) and Cap4 (PLA) underwent extensive cracking and bulk fragmentation.

Based on published studies of comparable blends, PLA is likely to form a continuous phase in Cap1 (PLA/PHBV, approximately 60/39 wt.%), although the PHBV phase may also be continuous, resulting in a co-continuous morphology [42]. Similarly, for Cap2 (PLA/PBAT, approximately 69/30 wt.%), PLA is expected to remain continuous, while PBAT may also form an interconnected phase, as Deng et al. [22] experimentally observed co-continuous morphologies in PLA/PBAT blends containing approximately 20–40 wt.% PBAT. Although known for its resistance to mesophilic AD, PLA underwent fragmentation, resulting in a significant loss of integrity in Cap1, Cap2 and Cap4. Similar behavior has been observed for PHBV, which degrades mainly by surface erosion [24,43].

For Cap3, excluding the inorganic fraction, the polymer composition corresponds to approximately 37/63 PLA/PBS. Based on phase-inversion behavior reported by Deng and Thomas [23] for comparable blends, PBS is likely to form the continuous polymer phase, with PLA present as dispersed domains. The low anaerobic biodegradability of PBS-based materials under mesophilic conditions has been reported by both Suarez Murcia et al. [15] and Zaborowska et al. [44]. The persistence of a PBS-rich continuous phase could therefore contribute to the preservation of Cap3’s macroscopic integrity after AD, despite the molecular changes observed. However, this interpretation remains hypothetical, as phase continuity was not directly characterized and may be influenced by the mineral filler and processing conditions.

In contrast, Cap4 consists of PLA without a second polymer component and therefore does not exhibit the phase morphology characteristic of immiscible polymer blends. Its extensive cracking and fragmentation after AD indicate that the presence of a second polymer phase is not required for substantial macroscopic deterioration under the investigated conditions. Nevertheless, the morphology and crystallinity of PLA itself may influence its susceptibility to hydrolysis. These phase-morphology interpretations are based on published studies of comparable blends rather than direct measurements of phase continuity or interfacial adhesion in the investigated capsules.

Overall, the cracking and fragmentation induced by AD may increase the surface accessible to water and microorganisms, thereby potentially enhancing biodegradation during subsequent composting [14,45]. 

### 3.3. Molecular Weight and Thermal Stability Changes

Size exclusion chromatography (SEC) performed on the recovered polymer residues before and after mesophilic AD showed a significant reduction in the number-average molecular weight (Mn) for all capsules (Table 2). The decrease ranged from a factor of 2 for Cap3 and Cap4 to factors of 5 and 8 for Cap1 and Cap2, respectively. This molecular change was consistent with thermogravimetric data: the PLA degradation peak (T_max_) shifted by 33 to 58 °C toward lower temperatures, consistent with chain scission, whereas PHBV, PBAT, and PBS exhibited no major changes (Table 2, Figure S3). The pronounced Mn decrease for Cap1 and Cap2 was accompanied by higher dispersity index values, indicating a broader molecular weight distribution with the coexistence of short, hydrolyzed PLA chains and longer, less affected chains assumed to correspond mainly to PHBV or PBAT. These differences suggest that the degradation behavior of the investigated materials depends on their overall formulation, including the possible influence of polymer-phase organization. The macroscopic and SEM observations reveal distinct morphological responses, with pronounced surface deterioration in Cap1, extensive cracking and fragmentation in Cap2 and Cap4, and preservation of the macroscopic integrity of Cap3 despite molecular changes. As each formulation was represented by a single commercial product, these differences cannot be attributed solely to the presence of PHBV, PBAT or PBS. Other material-specific characteristics, including initial molecular properties, crystallinity, additives, fillers, processing history and phase morphology, may also contribute to the observed behavior. Comparisons among capsules should therefore be interpreted at the level of the complete commercial formulations rather than as controlled comparisons of individual polymer components.

However, the observed decrease in PLA molecular weight demonstrates chain scission but does not, by itself, identify its mechanism. Abiotic hydrolysis is a plausible contributor, since PLA has been shown to undergo hydrolytic ester-bond cleavage in aqueous environments even below its glass-transition temperature [37]. Low-molecular-weight degradation products may subsequently be assimilated by the anaerobic microbial community, as suggested by previous studies reporting methane production from PLA-based materials [27,31]. The prolonged exposure to the aqueous AD medium (105 days at 38 °C, initial pH ≈ 8) was consistent with conditions allowing PLA hydrolysis. A microbial contribution, including possible extracellular enzymatic activity, cannot be excluded. However, in the absence of microbial characterization and a sterile abiotic control under comparable conditions, the respective contributions of abiotic hydrolysis and biologically mediated processes cannot be quantified.

DSC analysis revealed formulation-dependent changes in crystallinity following mesophilic AD (Table 3 and Figure S4). In Cap1, the initially predominantly amorphous PLA became crystalline after AD, consistent with hydrolysis-induced crystallization previously reported by Limsukon et al. [20]. The crystallinity degree of the PHBV phase did not change, which could be consistent with a surface erosion process leaving behind a bulk material with merely changed properties [24]. In Cap2, the PLA crystallinity increased after AD, whereas the PBAT contribution to the melting enthalpy remained low, consistent with its predominantly amorphous nature [20]. In the thermograms of Cap3, the cold crystallization peak observed at 90 °C, both before and after AD, cannot be unambiguously assigned to either PLA or PBS without complementary X-ray diffraction analysis. Therefore, Table 3 reports the minimum and maximum degrees of crystallinity for each polymer phase, calculated by alternatively assigning the cold crystallization peak to PLA or PBS. Despite the uncertainty in the absolute crystallinity values, the results indicate that the crystallinity of both polymer phases remained largely unchanged after AD. This observation is consistent with the preservation of its macroscopic integrity and its limited biodegradation during mesophilic AD (Figure 1 and Figure 2; Table 1). This behavior may be related to a possible PBS-continuous morphology, with PLA forming the dispersed phase [23], although this phase organization was not directly characterized. The crystallinity degree of PLA in Cap 4 slightly decreased after AD, although the difference remained within experimental uncertainty, suggesting that the hydrolysis process was not restricted to the amorphous phase. These results indicate that molecular weight reduction does not necessarily lead to an increase in crystallinity under mesophilic AD conditions.

Overall, the results suggest that PLA is likely the most hydrolysis-sensitive component under mesophilic AD, in line with previous findings [12,13]. Both studies reported preferential degradation of the PLA fraction in PBAT/PLA materials under anaerobic digestion, consistent with the lower anaerobic biodegradability of PBAT reported by Yu et al. [46].

### 3.4. Effect of AD Pre-Treatment on Composting Biodegradation Kinetics

Aerobic biodegradation experiments were performed under industrial composting conditions on residues recovered after AD and compared with untreated capsules (Figure 3). The combined AD–composting sequence markedly accelerated biodegradation of PLA-, PLA/PHBV-, and PLA/PBAT-based capsules, reducing the half-degradation time (*k*) by a factor of 2.3–3.7 (Table S1, SI), with statistically significant differences for Cap1, Cap2, and Cap4 (*p* < 0.05). Interestingly, anaerobic biodegradability alone did not appear to predict the subsequent acceleration of composting: despite their different BI values (59% for Cap1 and 29% for Cap4), both materials exhibited markedly faster biodegradation during subsequent composting. This indicates that BI alone is insufficient to explain the effect of AD on subsequent composting and suggests that the properties of the residual material after AD are also important. The AD residues of Cap1, Cap2, and Cap4 achieved complete biodegradation without a lag phase, whereas untreated samples required longer times to reach similar levels. In contrast, Cap3 (PBS/PLA) showed a smaller, non-significant reduction in half-degradation time (1.7-fold; *p* = 0.0611), despite measurable molecular changes after AD.

These results confirm that materials entering composting after AD fully biodegrade under industrial conditions, consistent with the well-established aerobic biodegradability of PLA, PHBV, PBAT, and PBS [9,47]. The faster composting biodegradation observed after AD was associated with the molecular and morphological changes induced during the pretreatment, but these observations do not establish a unique causal mechanism. In addition to PLA chain scission, AD caused a loss of material integrity, macroscopic fragmentation and marked surface morphological changes (Figure 2). Fragmentation is expected to increase the material surface accessible to water and microorganisms during subsequent composting and may therefore contribute to the shorter half-degradation times. Other AD-induced modifications, including changes in crystallinity, phase morphology and preferential degradation of individual components, may also contribute to the enhanced biodegradation.

Although the composting step in integrated-composting systems generally operates at slightly lower peak temperatures and for shorter durations than standalone composting, owing to the vegetal nature of the waste mixed with digestate, the accelerated degradation observed here likely compensates for these less favorable conditions. The synergy between AD and industrial composting thus enhances the efficiency of organic recycling by promoting faster conversion of PLA-rich residues. More fundamentally, these results demonstrate that the degradation behavior of commercial compostable formulations cannot be inferred from that of the constituent polymers considered individually. Rather, it is governed by the polymer formulation, phase organization and the resulting structural evolution during successive biological treatments.

## 4. Conclusions

This study demonstrates that mesophilic anaerobic digestion effectively enhances the subsequent composting biodegradation of PLA-based packaging materials. Under the mesophilic AD conditions investigated, PLA underwent chain scission, resulting in molecular weight reduction and material fragmentation. These AD-induced structural changes accelerated biodegradation kinetics during industrial composting by suppressing the initial lag phase and reducing the composting half-time by a factor of 2.3–3.7. In contrast, PBS-rich formulations were only marginally affected.

Overall, coupling AD and composting offers a promising integrated strategy for managing PLA-based packaging co-collected with biowaste. By accelerating biodegradation kinetics, this combined approach enhances the compatibility of compostable packaging with the shorter composting cycles typically implemented after AD. More fundamentally, this study demonstrates that the degradation behavior of commercial compostable formulations cannot be inferred from the behavior of the constituent polymers considered individually but is governed by polymer formulation and the structural changes occurring during successive biological treatments. Future studies should assess whether comparable structural modifications occur under shorter mesophilic retention times closer to operational conditions and under thermophilic AD, as well as for a broader range of commercial compostable formulations, with particular attention to hydrolysis kinetics and blend phase morphology. In an integrated AD–composting process, digestate containing partially degraded polymer residues would be directed to subsequent composting. However, the potential persistence of residual microplastics, the ecotoxicity and quality of the final compost, and the economic feasibility of the combined process remain to be evaluated under representative industrial conditions.

## Figures and Tables

**Figure 1:**
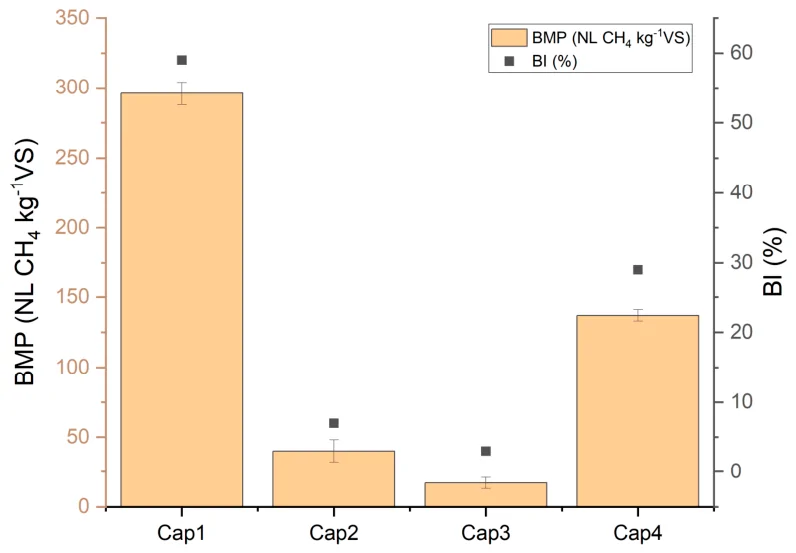
Biochemical Methane Potential (BMP) and Biodegradability Index (BI) of compostable coffee capsules after 105 days of mesophilic anaerobic digestion (38 °C): Cap1 = PLA/PHBV, Cap2 = PLA/PBAT, Cap3 = PBS/PLA, Cap4 = PLA. Error bars indicate standard deviation (*n* = 3).

**Figure 2:**
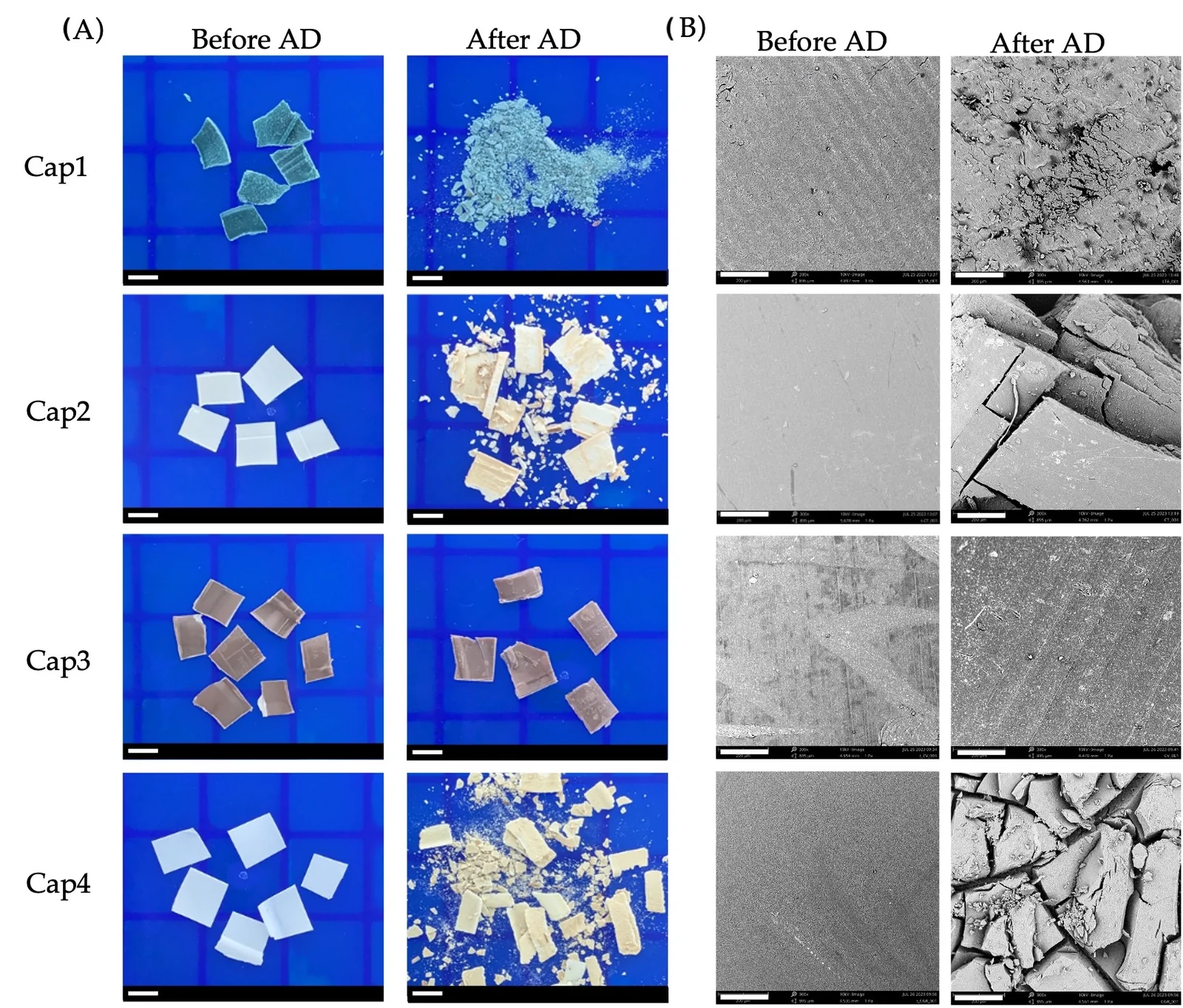
Macroscopic appearance (**A**) and scanning electron micrographs (**B**) of compostable coffee capsules before and after 105 days of mesophilic anaerobic digestion (AD). Cap1 = PLA/PHBV, Cap2 = PLA/PBAT, Cap3 = PBS/PLA, and Cap4 = PLA. Scale bars: 5 mm for macroscopic images (**A**) and 200 µm for SEM images (**B**).

**Figure 3:**
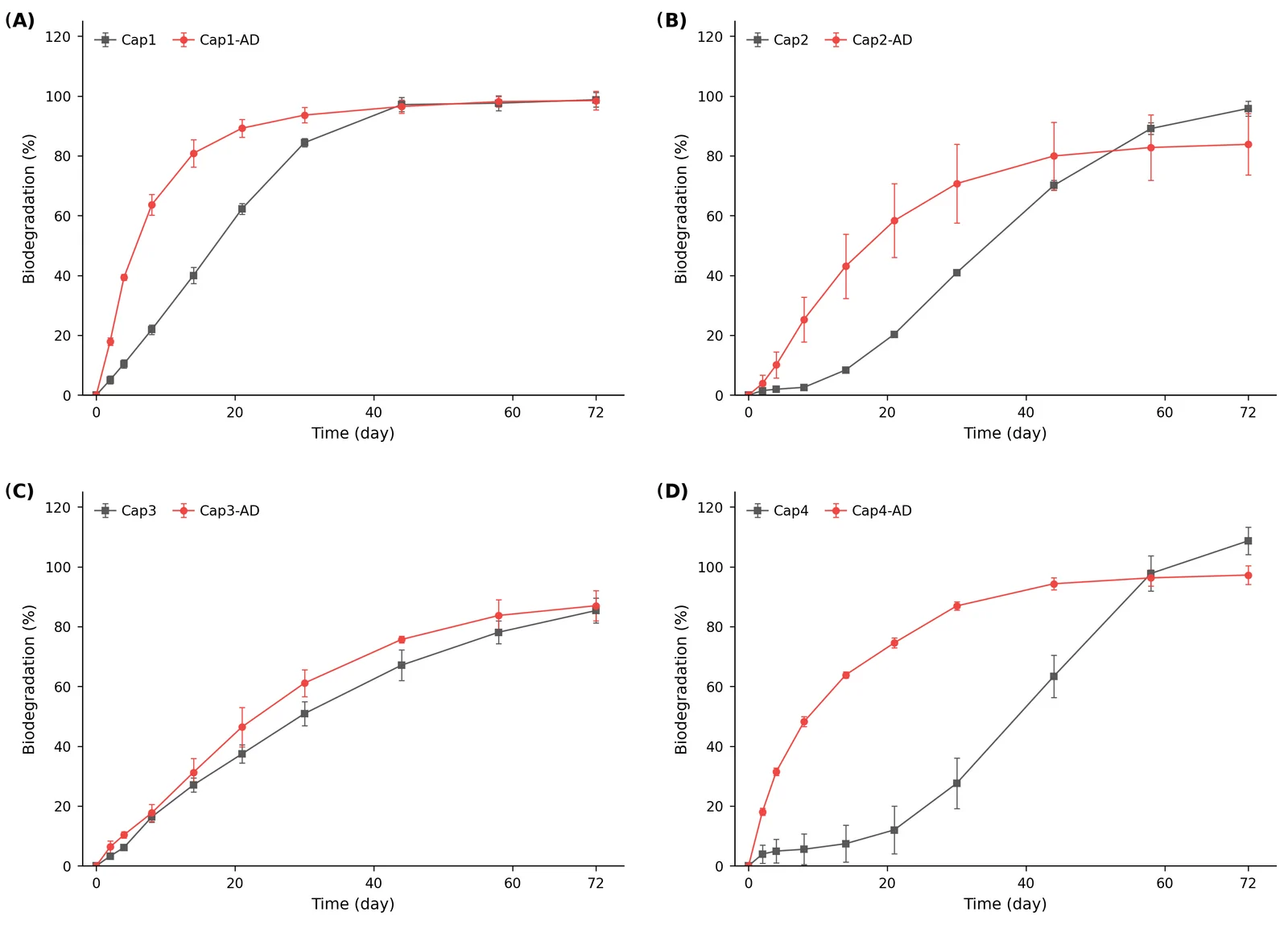
Biodegradation kinetics of compostable coffee capsules under industrial composting conditions. Solid line = untreated capsules; dashed lines = AD-residues with (**A**) Cap1 = PLA/PHBV, (**B**) Cap2 = PLA/PBAT, (**C**) Cap3 = PBS/PLA, (**D**) Cap4 = PLA. Experimental data are presented as mean ± standard deviation (*n* = 3).

**Table 1:** Composition of compostable coffee capsules before and after mesophilic anaerobic digestion. Polymer mass fractions (wt.%) were determined by thermogravimetric analysis (TGA), with polymer identities confirmed by ^1^H NMR spectroscopy. Values are means ± SD (*n* = 3).

Sample	PLA (wt.%)	PHBV (wt.%)	PBAT (wt%)	PBS (wt.%)	Ash (wt.%)
Cap1	59.9 ± 2.7	39.0 ± 2.1	—	—	1.1 ± 0.5
Cap1-AD residue	70.6 ± 3.1	25.8 ± 1.9	—	—	3.6 ± 1.7
Cap2	68.7 ± 2.1	—	29.7 ± 1.8	—	1.6 ± 0.4
Cap2-AD residue	55.4 ± 2.4	—	42.0 ± 2.3	—	2.6 ± 0.6
Cap3	30.3 ± 1.7	—	—	52.0 ± 2.3	17.7 ± 2.3
Cap3-AD residue	25.8 ± 2.0	—	—	56.3 ± 2.5	17.9 ± 2.7
Cap4	98.4 ± 1.9	—	—	—	1.6 ± 0.7
Cap4-AD residue	97.2 ± 1.8	—	—	—	2.7 ± 1.0

**Table 2:** Number-average and weight-average molecular weights (*M_n_* and *M_w_*), dispersity index (ĐM) determined by size exclusion chromatography (SEC) in chloroform at 30 °C, and temperatures of maximum mass-loss rate (T_max_) determined from derivative thermogravimetry (DTG) curves obtained by TGA under a nitrogen atmosphere (10 °C/min) for polymers extracted from coffee capsules before and after anaerobic digestion.

	Average Molecular Weight			T_max_ Temperature			
Sample	*M_n_* (kDa)	*M_w_* (kDa)	ĐM	PLA (°C)	PHBV (°C)	PBAT (°C)	PBS (°C)
Cap1	66.7 ± 2.3	160.5 ± 3.9	2.40	344 ± 3	292 ± 3	—	—
Cap1-AD	12.8 ± 1.1	72.4 ± 2.9	5.63	286 ± 2	286 ± 2	—	—
Cap2	55.3 ± 2.8	126.0 ± 3.4	2.28	349 ± 1	—	397 ± 2	—
Cap2-AD	6.7 ± 0.9	38.4 ± 1.8	5.77	292 ± 2	—	400 ± 2	—
Cap3	45.6 ± 2.1	106.1 ± 3.1	2.34	348 ± 2	—	—	392 ± 3
Cap3-AD	24.9 ± 1.8	51.50 ± 1.9	2.07	315 ± 2	—	—	396 ± 2
Cap4	100.3 ± 3.2	187.6 ± 5.0	1.87	361 ± 3	—	—	—
Cap4-AD	51.2 ± 1.8	126.0 ± 4.1	1.82	319 ± 2	—	—	—

**Table 3:** Melting enthalpies (Δ*H*_m_), cold crystallization enthalpies (Δ*H*_cc_), and degree of crystallinity (*X*_c_) of the constituent polymers before and after mesophilic anaerobic digestion.

	Δ*H_m_* (J/g)				Δ*H_cc_* (J/g)	X_c_ (%)			
Sample	PLA	PHBV	PBAT	PBS		PLA	PHBV	PBAT	PBS
Cap1	2.5 ± 1	14 ± 1	—	—	—	4 ± 2	25 ± 2	—	—
Cap1-AD	39 ± 3	10 ± 1	—	—	—	59 ± 7	26 ± 5	—	—
Cap2	24 ± 1	—	2.7 ± 2	—	—	38 ± 2	—	8 ± 7	—
Cap2-AD	25 ± 3	—	0.8 ± 1	—	—	49 ± 3	—	2 ± 1	—
Cap3 *	15 ± 3	—	—	41 ± 3	4	23–38	—	—	71–78
Cap3-AD *	12 ± 3	—	—	47 ± 3	6	24–47	—	—	65–76
Cap4	38 ± 2	—	—	—	—	41 ± 3	—	—	—
Cap4-AD	32 ± 3	—	—	—	—	35 ± 4	—	—	—

* For Cap3 and Cap3-AD, melting enthalpies (Δ*H_m_*) are reported without correction for cold crystallization (Δ*H_cc_* = 4 and 6 J/g, respectively). As the cold-crystallization peak near 90 °C could not be assigned unambiguously to PLA or PBS, crystallinity ranges (*X_c_*) were calculated by subtracting Δ*H_cc_* from the melting enthalpy of either polymer (minimum *X_c_*) or assigning it to the other polymer (maximum *X_c_*).

## Data Availability

Data will be made available upon request.

## Supplementary Materials

The following supporting information can be downloaded at: https://www.mdpi.com/article/10.3390/polym18212691/s1, Figure S1: ATR-FTIR spectrum of Cap3 (PBS/PLA) before anaerobic digestion; Figure S2: Cumulative specific methane production during 105 days of mesophilic anaerobic digestion (38 °C) of the four commercial compostable coffee capsules; Figure S3: Thermogravimetric analysis and derivative thermogravimetric curves of coffee capsules before and after anaerobic digestion; Figure S4: DSC thermograms of compostable coffee capsules before and after anaerobic digestion; Table S1: Hill model parameters (Deg_max_, k, n) describing biodegradation kinetics of compostable coffee capsules before and after mesophilic AD under industrial composting conditions (58 ± 1 °C). Parameters derived from CO_2_ evolution data.
