# Supplementary material for: Mesophilic Anaerobic Digestion as a Pre-Treatment to Enhance the Composting Biodegradation of PLA-Based Compostable Packaging
Source: Polymers (Basel). 2026 Oct 30;18(21):2691. doi: 10.3390/polym18212691 (PMC13766034; doi:10.3390/polym18212691)
Supplement: Supplementary file 1 [file polymers-18-02691-s001.zip › polymers-4602877-supplementary.pdf]

Supplementary Material - Polymers

Mesophilic Anaerobic Digestion as a Pre-treatment to Enhance the Composting Biodegradation of PLA-based Compostable Packaging

Gaël Huet[a], Julie Lamarque[b,e], Florian Monlau[b], Valentin Puchelle[c], Cecilia Sambusiti[d], Juliana Catalina Suarez Murcia[b,e], Sandra Domenek[f], Emmanuelle Gastaldi[a,]

[a] UMR IATE, Université de Montpellier, INRAE, L'institut Agro Montpellier, 34000 Montpellier, France

[b] TotalEnergies, OneTech R&D, PERL – Pôle Economique 2, 64170, Lacq, France

[c] TotalEnergies, OneTech Belgium, Feluy – Zone Industrielle C, 7181, Seneffe, Belgium

[d] TotalEnergies, OneTech R&D, CSTJF – Avenue Larribau, 64018, Pau, France

[e] UMR IPREM, Université de Pau et des Pays de l'Adour, CNRS, 64053 Pau, France

[f] UMR SayFood, Université Paris-Saclay, INRAE, AgroParisTech, 91120, Palaiseau, France

Corresponding authors: Emmanuelle.gastaldi@umontpellier.fr; florian.monlau@totalenergies.com

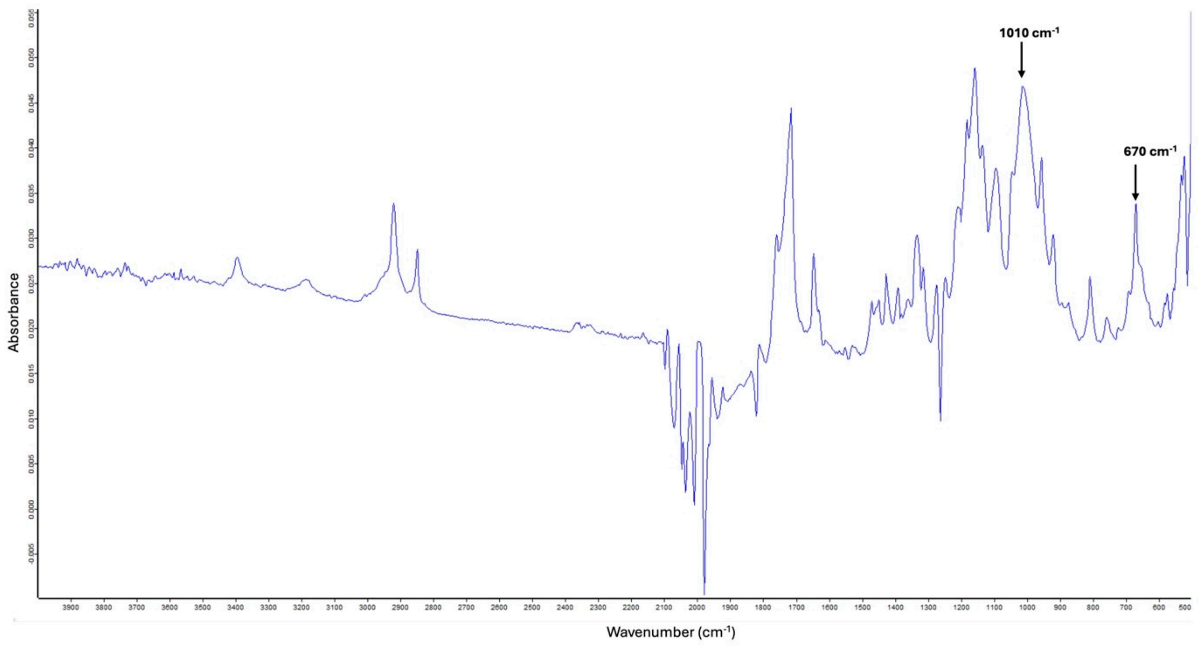

Figure S1. ATR-FTIR spectrum of Cap3 (PBS/PLA) before anaerobic digestion.

The spectrum was acquired using a Perkin Elmer FTIR spectrometer in ATR mode and processed using OPUS® software (Bruker, v8.2.28). The absorption bands at approximately 670 and 1010 cm^{-1} are consistent with silicate vibrations characteristic of talc [30].

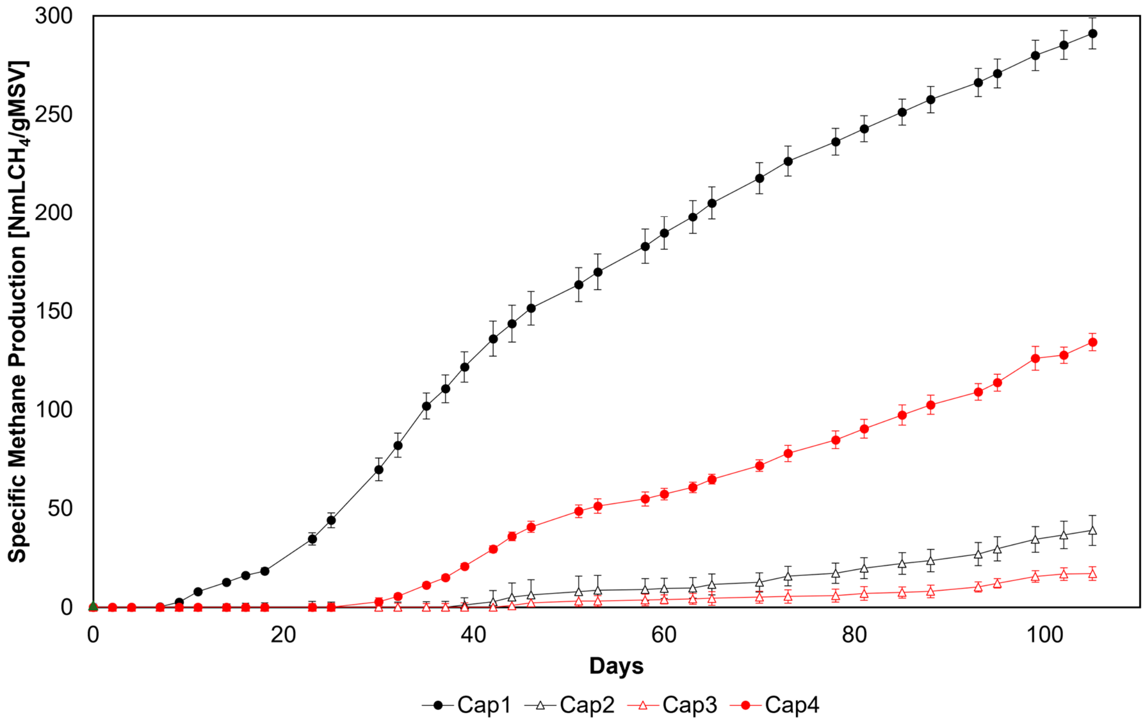

Figure S2. Cumulative specific methane production during 105 days of mesophilic anaerobic digestion (38 °C) of the four commercial compostable coffee capsules. Experiments were performed in triplicate (n = 3), and error bars represent standard deviations.

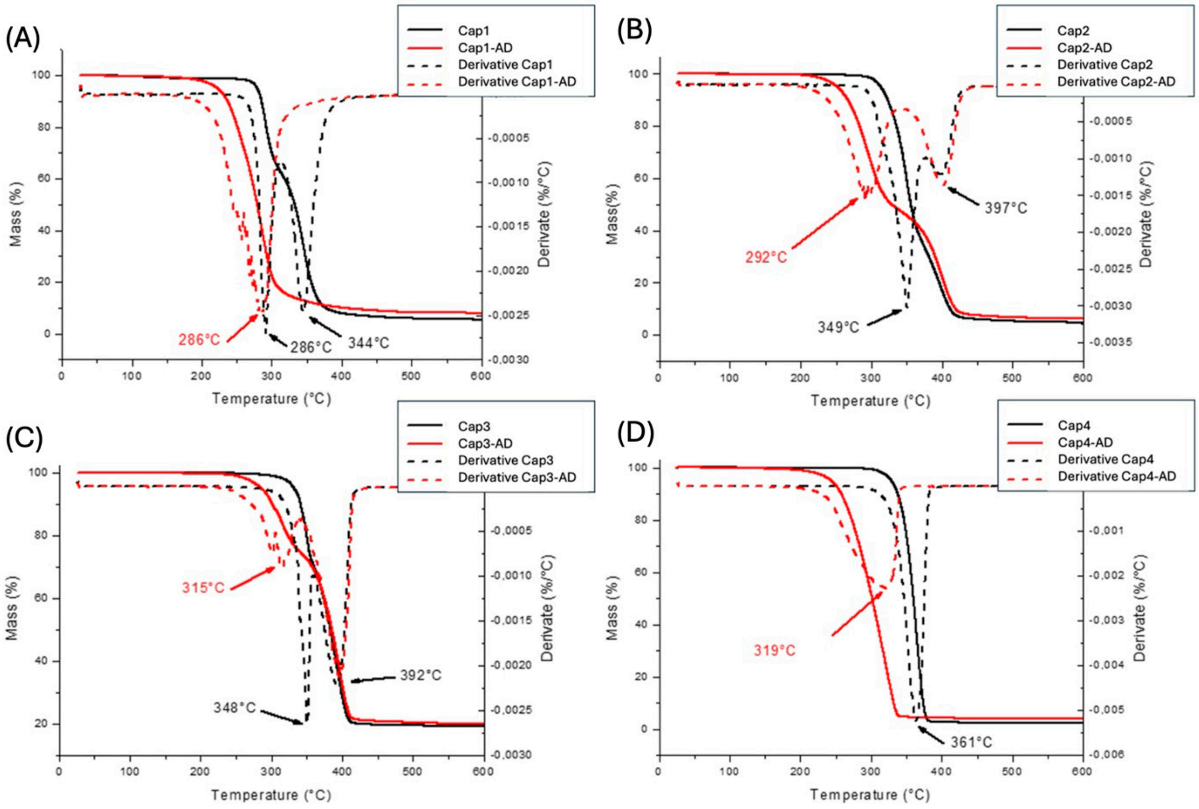

Figure S3. Thermogravimetric analysis (TGA) and derivative thermogravimetric curves (DTG) of coffee capsules before (black line) and after anaerobic digestion (red line); (A) Cap1 = PLA/PHBV, (B) Cap2 = PLA/PBAT, (C) Cap3 = PBS/PLA, (D) Cap4 = PLA.

The decrease in PLA thermal stability after AD (ΔT_{max} = −33 to −58 °C) is consistent with polymer chain scission.

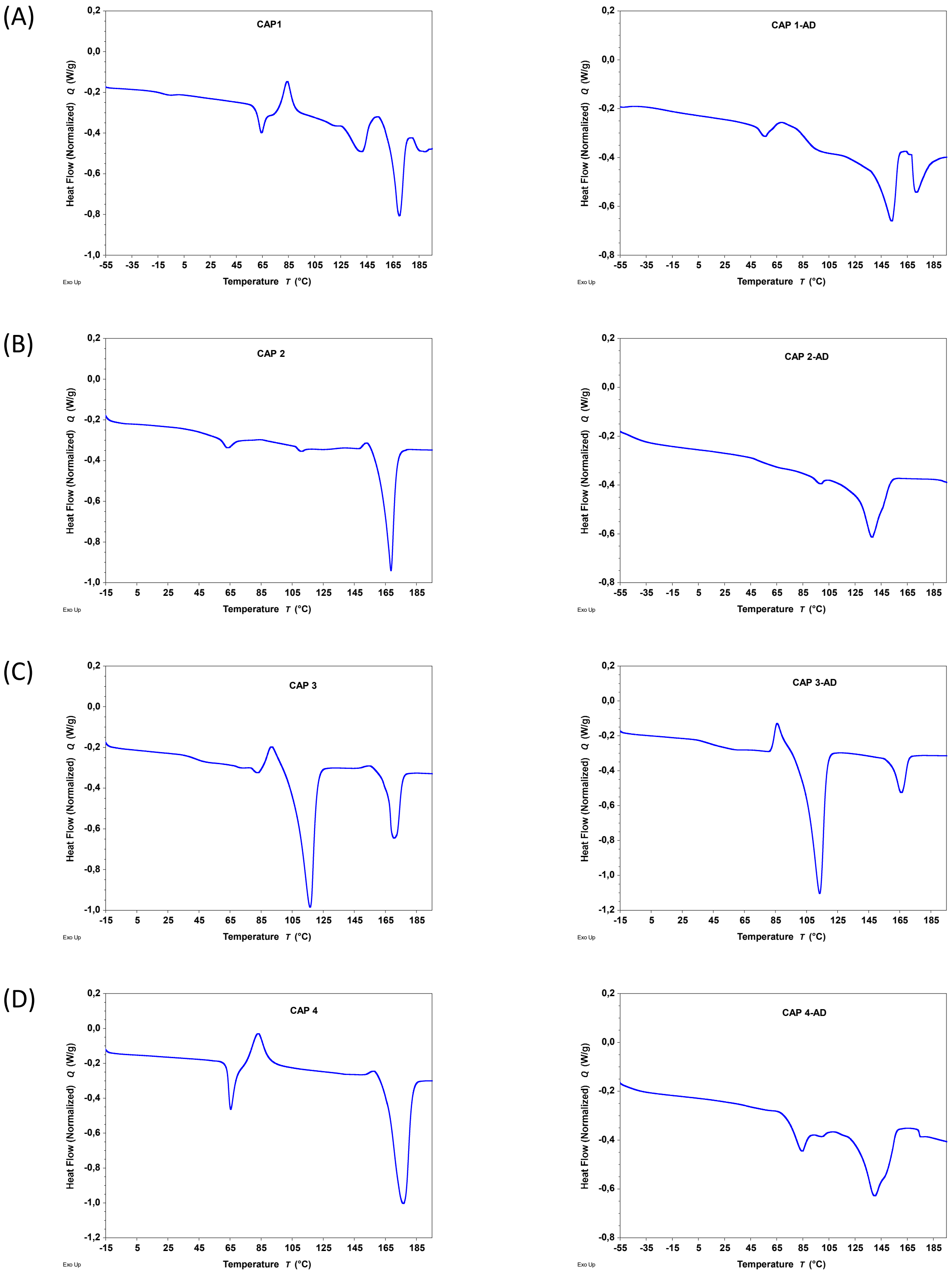

Figure S4. DSC thermograms of compostable coffee capsules before and after anaerobic digestion (AD): (A) Cap1 (PLA/PHBV) and Cap1-AD; (B) Cap2 (PLA/PBAT) and Cap2-AD; (C) Cap3 (PBS/PLA) and Cap3-AD; (D) Cap4 (PLA) and Cap4-AD. Blue lines represent the first heating scans.

Table S1. Hill model parameters (*Deg_{max}*, *k*, *n*) describing biodegradation kinetics of compostable coffee capsules before and after mesophilic AD under industrial composting conditions (58 ± 1 °C). Parameters derived from CO_2 evolution data.

Sample	Hill parameters				
	Deg_{max} (%)	*k* (day)	*n*	R^2	p-value (*k*)
Cap1	108.86 ± 3.99	17.26 ± 0.05	1.90 ± 0.12	0.994	0.0006
Cap1-AD residue	101.25 ± 3.13	5.56 ± 0.52	1.50 ± 0.19	1.000	
Cap2	105.60 ± 4.74	34.47 ± 0.97	2.95 ± 0.16	0.999	0.0017
Cap2-AD residue	91.42 ± 10.35	14.91 ± 2.37	1.66 ± 0.05	0.999	
Cap3	125.83 ± 9.70	40.64 ± 8.53	1.25 ± 0.08	0.999	0.0611
Cap3-AD residue	105.60 ± 12.80	24.35 ± 6.19	1.47 ± 0.17	0.996	
Cap4	120.78 ± 12.76	41.42 ± 0.70	3.76 ± 1.09	0.991	0.0012
Cap4-AD residue	115.68 ± 10.26	11.19 ± 2.49	1.01 ± 0.12	0.998	

Parameters are expressed as mean ± standard deviation from independent Hill fits of three reactor curves (n = 3). All available measured time points, including day 91 where present, were used for parameter fitting. Differences in k between untreated and AD-pretreated samples were assessed using Welch's t-test, with a significance threshold of $p < 0.05$.
